# Schiff‐bases for sustainable battery and supercapacitor electrodes

**DOI:** 10.1002/EXP.20210128

**Published:** 2021-12-28

**Authors:** Erik Troschke, Martin Oschatz, Ivan K. Ilic

**Affiliations:** ^1^ Center for Energy and Environmental Chemistry Jena (CEEC Jena) Institute for Technical Chemistry and Environmental Chemistry, Friedrich‐Schiller‐University Jena Jena Germany; ^2^ Center for Nano Science and Technology@PoliMi Istituto Italiano di Tecnologia Milan Italy

**Keywords:** batteries, carbons, Schiff‐bases, supercapacitors, sustainability

## Abstract

The quest for more efficient ways to store electrical energy prompted the development of different storage devices over the last decades. This includes but is not limited to different battery concepts and supercapacitors. However, modern batteries rely on electrochemical principles that often involve transition metals which can for instance suffer from toxicity or limited availability. More sustainable alternatives are needed. This sparked the search for organic electrode materials. Nevertheless, compared to their inorganic counterparts, organic electrode materials remain less intensely investigated. Besides the often more complicated electrochemical principles, one likely reason for that are the complex synthetic skills required to develop novel organic materials. Here we review materials synthesized by an old and comparably simple reaction from the field of organic chemistry, namely Schiff‐base formation. This reaction can often yield materials under relatively mild conditions, making them especially interesting for the formation of sustainable electrodes. The main weakness of Schiff‐base materials, susceptibility to hydrolysis, is of limited concern in most of the battery systems as they mostly anyways require water‐free conditions. This review gives an overview of some selected nanomaterials obtained from Schiff‐base formation as well as their carbonized derivatives which are of interest for energy storage. Firstly, the general chemistry of Schiff‐bases is introduced, followed by an in‐depth survey of the most important breakthroughs in the formation of organic battery electrodes that involve materials based on Schiff‐base reaction. Lastly, an outlook considering the main hurdles as well as future perspectives of this research area is given.

## Graphical abstract

A Schiff‐base reaction is a condensation reaction between amine and carbonyl groups. It can be used to synthesize various organic materials. The precursors are often low‐cost, and the synthesis utilizes mild conditions without the use of toxic chemicals. Therefore, these materials gained significant attention for sustainable electrodes. In this review, we discuss their applicability for sustainable batteries and electrodes.

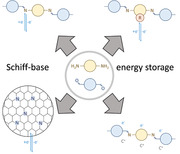

## 1 SUSTAINABLE ENERGY STORAGE

During the last decades, humankind became increasingly aware of its negative impact on the environment. It turned out unequivocally that anthropogenic activity changes nature and causes effects such as global warming. This prompted a shift from established, unsustainable, and carbon dioxide intense technologies based on fossil fuels to novel, sustainable, and carbon neutral technologies. Such alternatives must be primarily based on electric energy generated from renewable sources. However, electrochemical energy storage is rather difficult and special storage tools are needed. This triggered a broad research interest in storage devices such as batteries and supercapacitors.^[^ ^1^ ^]^

Batteries store energy by transferring electrons from a material with high reduction potential, a cathode, to a material with lower reduction potential, an anode, at a defined electrochemical potential. Subsequently, this energy can be released on‐demand as electrical energy at a stable potential. On the other hand, supercapacitors store electrical energy by transferring electrons from one conductive material to another. The electrodes usually consist of activated carbon with a high surface area. The either positively or negatively charged electrode surface gets stabilized by an electric double layer of mirror charges. The capacitive current obtained does not have a defined, but instead an altering potential. Batteries and supercapacitors are complementary electrochemical energy storage devices as batteries are suitable for applications where high energy density is needed while supercapacitors can achieve higher power densities.^[^ ^2^ ^]^

Lithium‐ion batteries, one of the most widespread types of commercial batteries, rapidly dominated the market after their introduction in the early 1990s. This battery type mostly uses cathodes based on heavy metals such as cobalt and thus produces a severe negative impact on the environment, not only during mining but also during disposal of old batteries.^[^ ^3^ ^]^ This prompted researchers to look for organic and less toxic replacements for these established cathode materials. Redox‐active organic polymers came up as a facile solution. They have low solubility in common electrolytes, hence enabling longer cycle life as compared to small organic molecules.^[^ ^4^, ^5^ ^]^ The theoretical capacity (*C*_t_) of a redox‐active polymer can be calculated as:

(1) $$\;C_{t}=\frac{zF}{M}$$

where *z* is the number of electrons transferred per repeating unit, *F* is the Faraday constant (26801 mAh mol^–1^), and *M* is the molar mass of the repeating unit.

Anodes of commercial lithium‐ion batteries are predominantly made from graphite – a material that is abundantly available. However, the constant desire for lighter devices led to the search for materials that can store more lithium per mass than graphite. Especially nitrogen‐doped carbon materials,^[^ ^6^, ^7^ ^]^ as well as organic polymers,^[^ ^5^ ^]^ hold great promise for that purpose. In addition, the natural scarcity of lithium, when compared to other alkali metals, prompted researchers to develop alternative battery concepts, such as sodium‐ion batteries.^[^ ^8^, ^9^, ^10^ ^]^ Unlike graphite,^[^ ^11^ ^]^ nitrogen‐doped carbons are suitable candidates as anodes in sodium‐ion batteries.^[^ ^12^, ^13^ ^]^ Moreover, nitrogen‐doped carbons are promising materials for supercapacitors, as nitrogen doping can impact the electric double‐layer formation through a variety of effects such as increased wettability and in some cases enhanced conductivity.^[^ ^14^, ^15^, ^16^ ^]^ For both classes of materials, a precise chemical architecture in combination with defined and controllable texture on a broad range of scales is needed for a successful technological implementation in electrochemical energy storage.

Both organic polymers with redox‐active functionalities as well as N‐doped carbons (after further carbonization of precursor polymers) can be formed employing a simple amine‐carbonyl condensation reaction – namely the Schiff‐base formation. The resulting polymeric materials contain imine bonds which can either be themselves part of a redox‐active moiety or can connect redox‐active moieties. These materials are remarkably versatile in terms of chemical properties as various functional groups can be simply introduced with the molecules that undergo condensation. Additionally, the high‐surface area of some Schiff‐base materials can enable a postsynthetic introduction of redox‐active moieties or can be useful for their application as supercapacitors. Furthermore, the often high nitrogen content of Schiff‐base polymers makes them suitable precursors for N‐doped carbons. On the one hand, the structure of the formed carbons can be tuned to match the desired application by changing the precursors and/or the carbonization method. On the other hand, the formation of imine bonds provides a rather defined structure of the polymeric networks which can thus follow a specific chemical pathway for further condensation at higher temperatures. By that, N‐doped carbon materials with comparably uniform bonding motives of the heteroatoms can be obtained.

This family of polymeric materials and the derived carbons have been intensely investigated by materials chemists and researchers from related areas over the recent years. It is the major idea behind this review article to give an overview over selected examples and to summarize the underlying phenomena and mechanisms from the viewpoint of a materials chemist. Schiff‐base formation is an intriguing reaction due to its simplicity as it can often proceed without any additional catalyst and toxic waste products, unlike other polymer‐forming reactions, such as radical polymerizations. In the second chapter, the mechanistic details of the Schiff‐base reactions will be discussed along with the historical background. Some of the most common types of the Schiff‐base materials found in the literature will be introduced and their applicability in energy storage technologies will be discussed. In the following chapters, the different ways to utilize Schiff‐base materials for electrochemical energy storage will be discussed. In the third and fourth chapter, selected examples of Schiff‐base materials as anodes and cathodes for alkali metal‐ion batteries will be given. In the fifth chapter, the discussion will focus on the way Schiff‐base networks (SNWs) and frameworks can be applied as electrode materials in supercapacitors, utilizing their high surface area for electric double‐layer formation. In the following chapter, the nitrogen‐doped carbonaceous materials obtained upon exposure of Schiff‐base materials at elevated temperatures will be discussed. Lastly, an outlook will be given concluding the most important rules to design Schiff‐base materials for electrochemical energy storage.

## 2 SCHIFF‐BASE REACTION AND MATERIALS

Since the discovery of the condensation reaction between primary amines and aldehydes, these reactions have attracted significant attention.^[^ ^17^ ^]^ This reaction was firstly observed by Hugo Schiff and reported in 1864, as a reaction between aniline and different aldehydes.^[^ ^18^, ^19^ ^]^ It was noted that it proceeds with the formation of water and an increase in temperature, pointing at a condensation mechanism. A special type of imines, that can be prepared as a reaction product of primary amines and aldehydes or ketones, is called Schiff‐bases, honoring its discoverer.

### 2.1 Formation of Schiff‐bases

**FIGURE 1:**
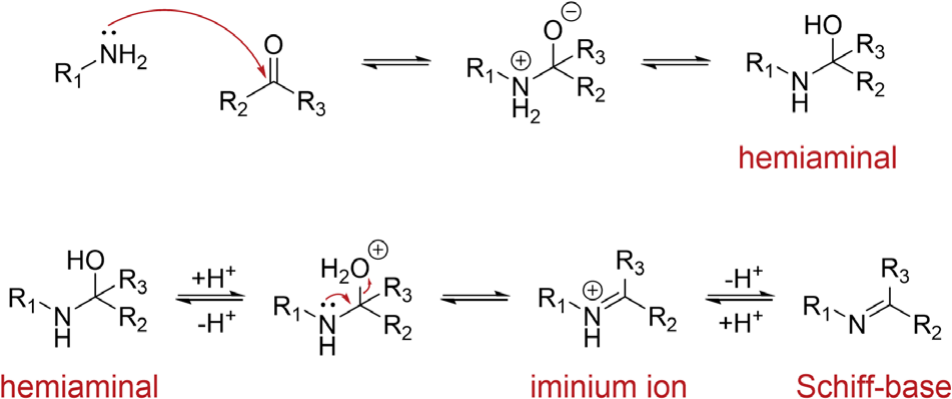
Mechanism of Schiff‐base formation

The reaction of amines with carbonyl groups starts with the nucleophilic attack of the amine to the carbonyl group (Figure 1). Upon proton transfer, a hemiaminal, which consists of a secondary amine and a hydroxyl group on the adjacent carbon atom, is formed. Protonation of the hydroxyl group enables the elimination of water and the iminium ion is formed. Deprotonation of the latter forms the imine, characterized by its carbon‐nitrogen double bond. While the second step, hemiaminal dehydration, is catalyzed by acidic conditions due to hemiaminal protonation, the first step, hemiaminal formation is hindered in acidic conditions as protonated primary amines are less nucleophilic. Therefore, the contributions of the individual reaction steps to the overall rate are strongly depending on the activity of protons during condensation.^[^ ^20^ ^]^

All the steps of the imine formation are reversible and represent the main obstacle in the applicability of Schiff‐base materials in many real‐world applications: imines can be hydrolyzed when exposed to an aqueous or humid environment. In many cases, the chemical equilibrium of aliphatic aldehydes or ketones and primary amines is shifted towards the reactants, and water removal from the reaction mixture is often needed. However, this is not the case for aromatic aldehydes, as they react easier than ketones.^[^ ^21^ ^]^ The reactivity of aldehydes can be ascribed to the combination of steric effects, as hydrogen is less bulky than alkyl chains, and electron density is lower on aldehyde carbon compared to ketone carbon. Furthermore, the reaction can be accelerated by many catalysts^[^ ^22^ ^]^ but this is out of the scope of this paper as the focus will be pointed on reactions without metallic catalysts.

As previously mentioned, many Schiff‐bases can easily form under rather mild conditions and without toxic organic solvents. Syntheses of Schiff‐bases in aqueous solution were frequently reported, however, with discrepancies regarding the obtained yields. It was observed that these discrepancies occur as Schiff‐bases predominantly form while water is being removed from the system during the drying step.^[^ ^23^ ^]^ Furthermore, it was noted that Schiff‐bases can be prepared using mechanochemistry, just by inducing a contact between the crystals of the precursors.^[^ ^24^ ^]^ Melt synthesis has also been reported, where one component in the mixtures of the precursors is heated at a temperature higher than the melting point of the other precursors.^[^ ^25^ ^]^

While there is a great variety of materials containing Schiff‐bases, from single molecules to complex networks, this review will focus on the polymeric materials that contain long chains or networks rich in Schiff‐base moieties. In the next chapter, the main considerations for the design of these materials will be discussed and the main representatives of this group of materials will be showcased.

### 2.2 Schiff‐base materials

While the imine bond is a strong chemical bond and can withstand harsh conditions when used in a water‐free environment, the application of Schiff‐base materials is often hindered due to their instability when getting in contact with water. This is why inherently water‐free alkali‐ion batteries are an attractive platform to apply materials containing imine bonds. However, not only pristine Schiff bases, but also carbonaceous materials derived from Schiff‐bases are promising materials for electrochemical energy storage.

Carbon materials have gained great attention as alkali‐metal battery anodes^[^ ^11^, ^28^, ^29^, ^30^ ^]^ and as electrodes for supercapacitors.^[^ ^31^, ^32^, ^33^ ^]^ Preparation of carbons at elevated temperatures requires starting materials that do not sublime or evaporate. For this reason, polymers are often utilized as they start to condense and carbonize before they evaporate. However, sustainable, naturally occurring polymers are in most cases poor in nitrogen, a heteroatom that is often used to improve the electrochemical properties of carbons. Doping of carbon with nitrogen is rather straightforward as the heteroatoms with comparable size can be incorporated into a framework of carbon atoms or attached to the surface and defect sites as functional groups. Organic chemistry holds a lot of inspiration to prepare such structures. Nitrogen generally changes the electrochemical properties of the carbon materials due to induced changes in the electron density distribution and band positions. The introduced polarity enhances the adsorption properties and the wetting ability of carbon materials.^[^ ^34^, ^35^, ^36^, ^37^, ^38^ ^]^ A notable example for a nitrogen‐containing sustainable polymer is chitin or chitosan, but even these precursors often result in carbons with modest nitrogen doping and barely controllable carbonization chemistry.^[^ ^39^, ^40^, ^41^ ^]^ By utilization of small molecules containing amine and carbonyl groups, the amine‐carbonyl condensation can be triggered, thus forming polymers that yield inherently N‐doped carbons due to a high density of imine‐linkages.

Schiff‐base polymers can be made when molecules containing multiple amine groups and molecules containing multiple carbonyl groups react,^[^ ^42^, ^43^ ^]^ or when a polymer containing one of these groups is brought to reaction with small molecules containing the other.^[^ ^44^, ^45^ ^]^ As Schiff‐bases contain carbon‐nitrogen double bonds, aromatic precursors can yield conjugated Schiff‐base polymers. Highly crosslinked conjugated Schiff‐base polymers, prepared by condensation of at least a triamine or a trialdehyde with a dialdehyde or a diamine, can form porous networks. In the following, a few examples of commonly encountered Schiff‐base frameworks and networks are given.

**FIGURE 2:**
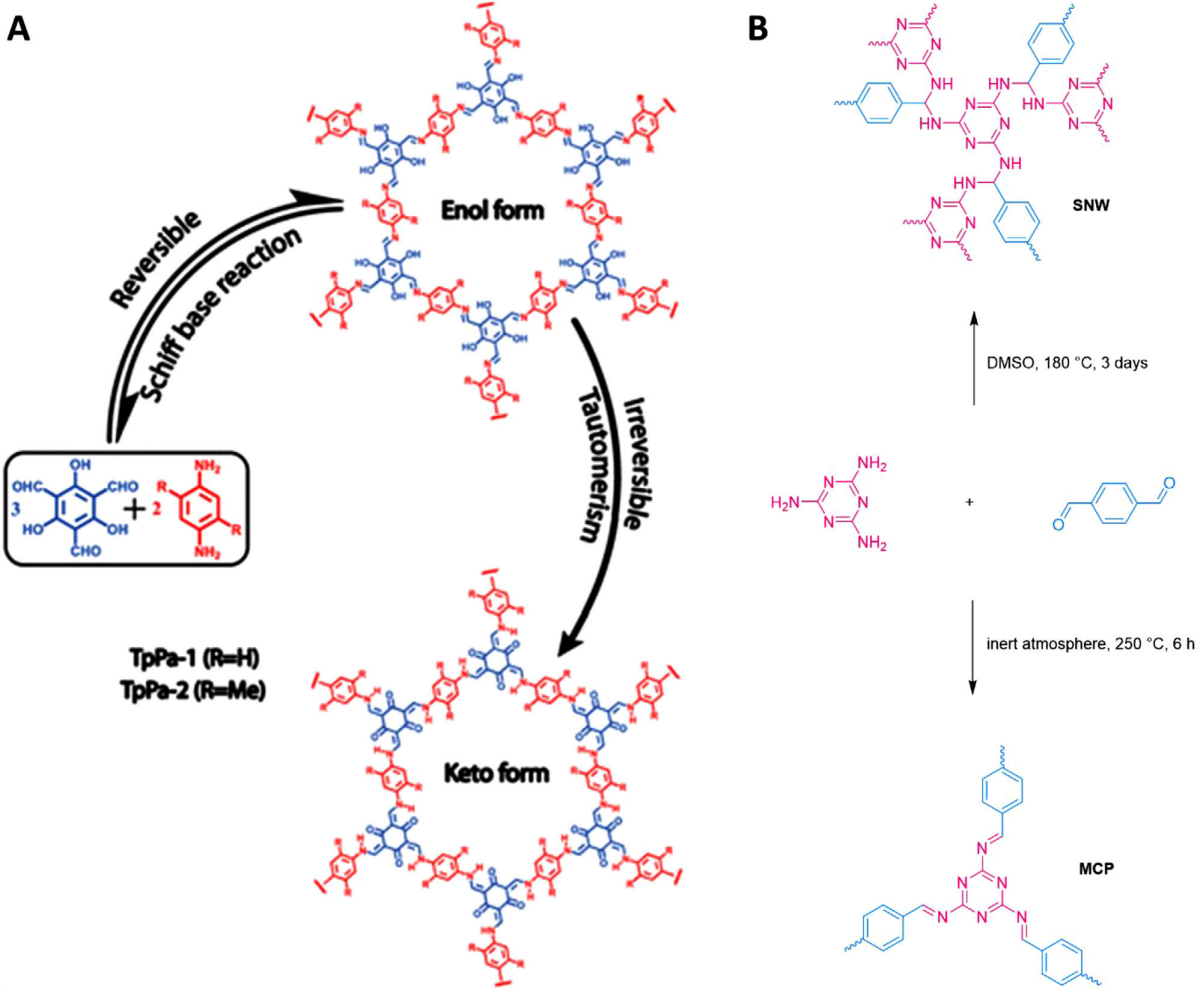
(A) Covalent organic frameworks synthesized using 1,3,5‐triformylphloroglucinol and aromatic diamines displaying subsequent irreversible tautomerization. Reproduced with permission.^[^ ^26^ ^]^ Copyright 2012, American Chemical Society. (B) Structures of a melamine‐based Schiff‐base network (SNW) and a melamine‐based conjugated polymer (MCP) synthesized from melamine and terephthalaldehyde, as proposed from the original works.^[^ ^25^, ^27^ ^]^

Covalent organic frameworks (COFs) are organic frameworks with individual organic building units connected through covalent bonds. The often‐observed crystallinity of COFs is a direct consequence of the reversibility of the reactions utilized for their preparation, which inherently limits the bond types available for their preparation.^[^ ^46^, ^47^, ^48^ ^]^ Yaghi et al. prepared the first COF by reversible condensation of diboric acid, forming boron‐oxygen six membered rings.^[^ ^49^ ^]^ Soon afterward, the same group synthesized the first COF based on Schiff‐base chemistry, thus significantly expanding the library of molecules available for COF synthesis.^[^ ^50^, ^51^ ^]^ Afterwards, Kuhn et al. reported nitrile trimerization reaction, where three nitrile groups form a triazine ring, for the formation of COFs, specifically called covalent triazine frameworks (CTFs).^[^ ^52^ ^]^ In this review, only COFs prepared using Schiff chemistry will be discussed. As mentioned, Schiff bonds show low stability in atmospheric conditions which greatly limits their applicability. Banerjee et al. proposed a solution for this problem by a combination of reversible and irreversible reactions. 1,3,5‐triformylphloroglucinol (Tp), in reaction with different diamines, forms COFs (TpCOFs) stable to hydrolysis as imine bonds are not present in the frameworks, since they undergo irreversible tautomerization. In consequence, the formed framework contains only the keto‐enamine form (Figure 2A).^[^ ^26^ ^]^ Although COFs for energy storage have been previously reviewed,^[^ ^53^, ^54^, ^55^, ^56^, ^57^, ^58^, ^59^ ^]^ this review will also include imine COFs but the focus will be given to the properties arising from their Schiff‐base nature, rather than their general COF character. In the review, COFs containing imine bonds will be abbreviated as “ImCOFs.”

As another example, melamine‐based SNWs are a class of materials prepared through solvothermal condensation of melamine and di/trialdehydes in dimethyl sulfoxide, without any catalyst (Figure 2B).^[^ ^27^ ^]^ The resulting amorphous networks are characterized by high surface areas of up to 1400 m^2^ g^–1^. Although the absence of carbonyl and amine bonds after polymerization reaction was witnessed, it was not possible to prove the existence of imine bonds (using IR spectroscopy) and thus the exact nature of these polymers remains unsolved to our knowledge. A comparable type of materials prepared from the same precursors is melamine‐based conjugated polymers (MCPs) (Figure 2B). MCPs are synthesized from the same precursors as SNWs but under solvent‐free conditions and under inert atmosphere at relatively low temperatures (150–250 °C for melamine‐terephtalaldehyde networks).^[^ ^25^ ^]^ The formed MCPs contain imines, as confirmed by ^13^C NMR and XPS measurements, while the absence of imine peaks in IR was ascribed to the overlap with the triazine peaks.

**FIGURE 3:**
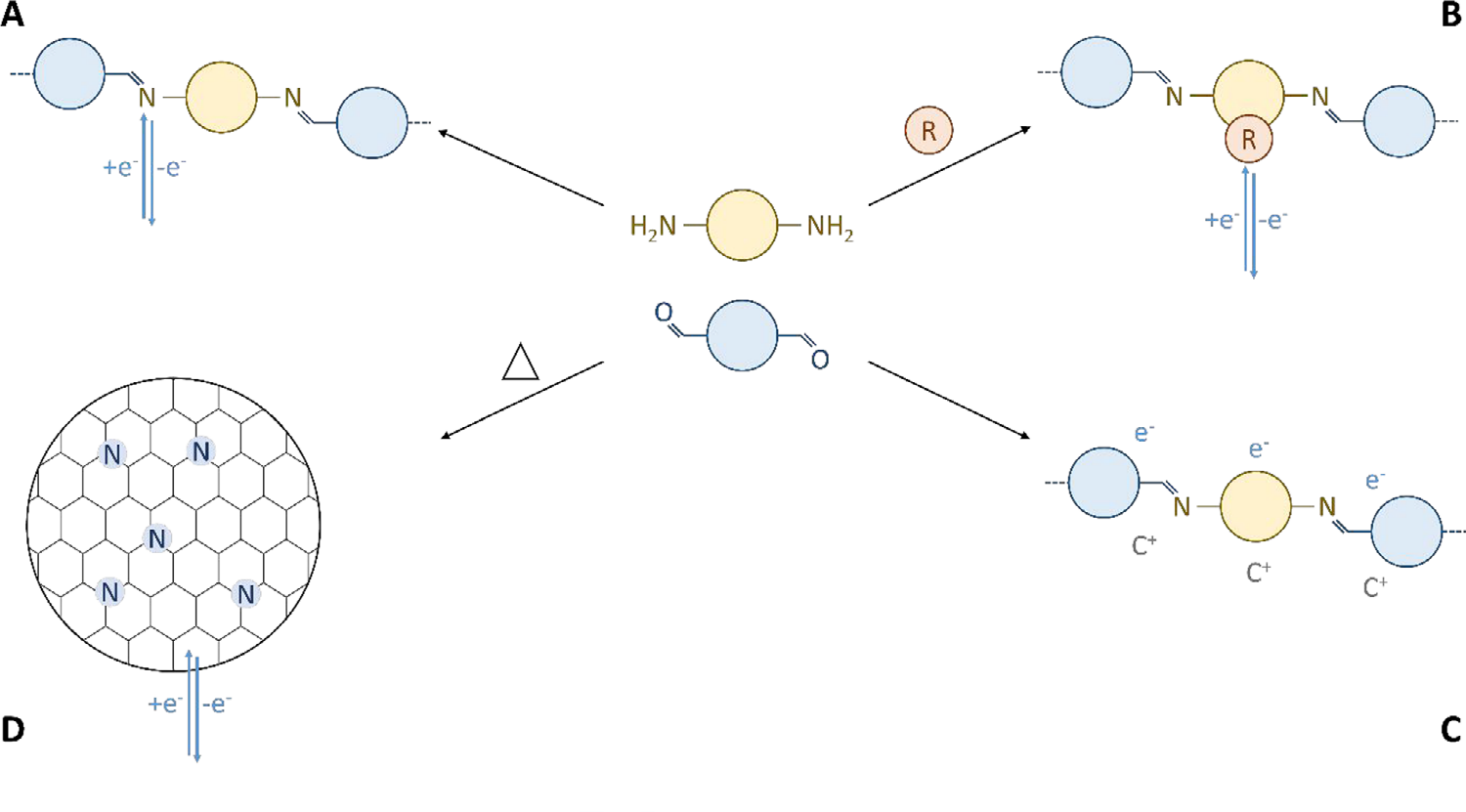
Different strategies to utilize Schiff‐base materials for electrochemical energy storage. (A) Materials containing Schiff‐bases are electroactive at low redox potentials making them attractive materials for organic anodes. (B) Schiff‐base materials can be utilized as host materials for different redox‐active species. (C) Microporous Schiff‐base networks and frameworks can be utilized as materials for supercapacitors. (D) Carbonized Schiff‐base materials can be utilized for supercapacitors and battery anodes

In the next chapters, the recent progress of the usage of Schiff‐base materials in electrochemical energy storage will be discussed. These materials were classified into 4 subcategories: materials that utilize redox reactivity of Schiff‐bases, materials that contain other redox‐active centers, microporous materials used as supercapacitors, and carbon materials derived from Schiff‐base materials (Figure 3).

## 3 SCHIFF‐BASES AND DERIVED GROUPS AS REDOX‐ACTIVE CENTERS

Early polarographic studies performed on conjugated Schiff‐base compounds showed reversible reactions occurring at low potentials in organic electrolytes.^[^ ^60^ ^]^ These studies recently inspired scientists to study the electrochemical behavior of Schiff‐bases in sodium‐ion (and subsequently lithium‐ion) systems and to evaluate these compounds as anodes for alkali‐ion batteries.^[^ ^61^ ^]^

### 3.1 Sodium storage

**FIGURE 4:**
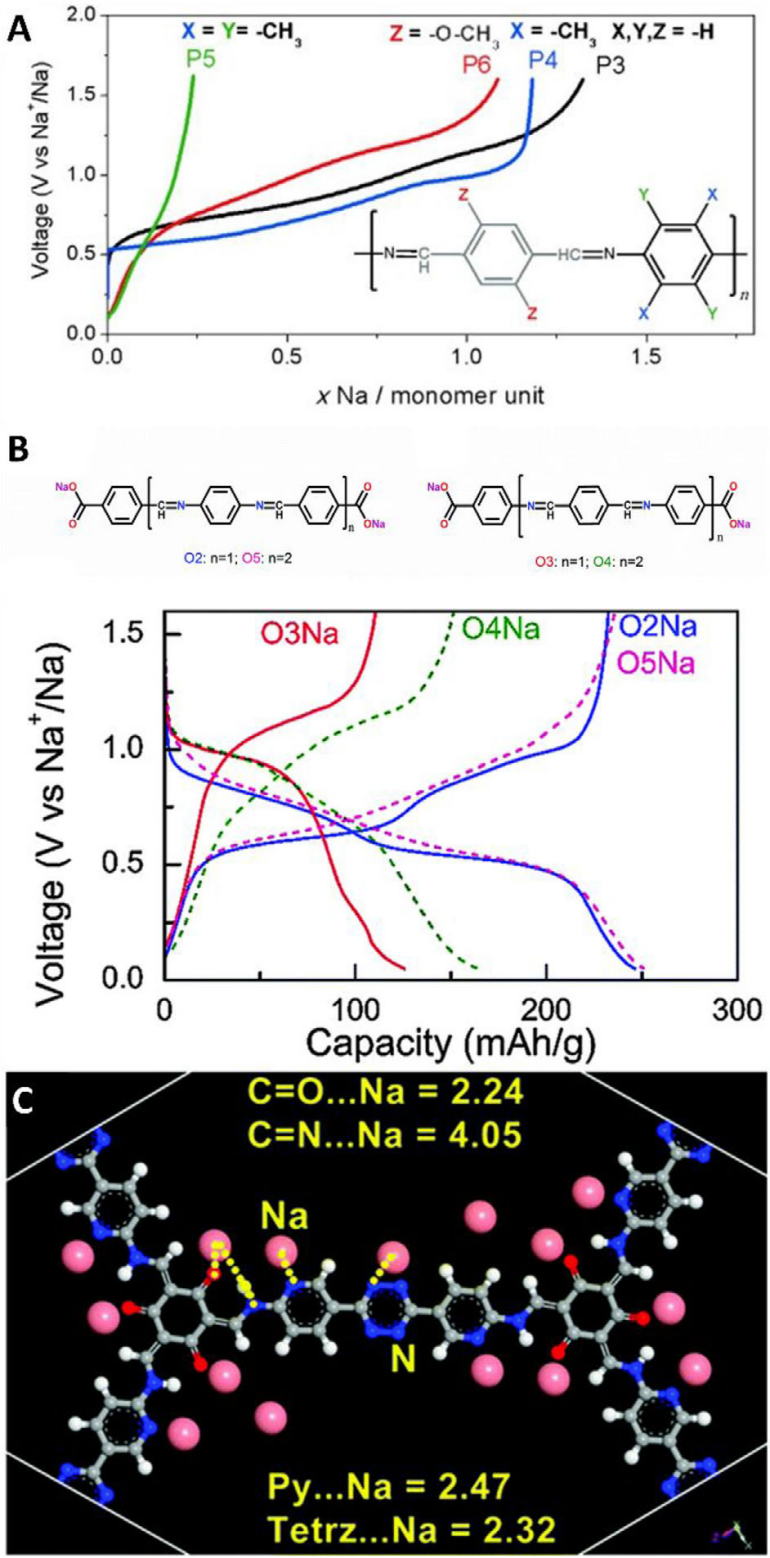
(A) Performance of conjugated Schiff‐base linked networks of terephthalaldehyde and *p*‐phenylenediamine with different substituents as anodes in sodium‐ion batteries. Reproduced with permission.^[^ ^61^ ^]^ Copyright 2014, Wiley‐VCH. (B) Oligomeric Schiff‐bases with sodium carboxylate end groups and their sodiation‐desodiation curves. Reproduced with permission.^[^ ^62^ ^]^ Copyright 2015, The Royal Society of Chemistry. (C) The DFT model of a sodiated TpCOF containing bipyridine and tetrazine groups. Reproduced with permission.^[^ ^63^ ^]^ Copyright 2020, The Royal Society of Chemistry

Armand et al. reported linear polymeric Schiff‐bases formed upon condensation of aromatic dialdehydes with aliphatic and aromatic diamines that showed promising results as anodes for sodium‐ion batteries, reaching capacities up to 350 mAh g^–1^ at 26 mA g^–1^.^[^ ^61^ ^]^ The Schiff‐bases were formed by azeotropic water removal in refluxing toluene and the reaction was confirmed with infrared spectroscopy. While all the conjugated polymeric Schiff‐bases, prepared from both aromatic dialdehydes and diamines, performed well as sodium‐ion anodes, not all non‐conjugated Schiff‐bases did. Much better performance was observed for the Schiff‐bases prepared from aromatic dialdehydes and aliphatic diamines as compared to aliphatic dialdehydes and aromatic diamines, suggesting the importance of ─N═CH─Ar─CH═N─ units for low‐voltage sodium storage. Furthermore, the effect of the functionalization of conjugated polymeric Schiff‐bases was studied in detail (Figure 4A). The addition of two methyl groups at the aromatic ring of diamine, or two methoxy groups at the aromatic ring of the dialdehyde resulted in insignificant changes in the performance of the material. However, the addition of four methyl groups to the aromatic diamine resulted in the material with much lower performance, due to the loss of planarity in the polymeric chain as a consequence of steric hindrance. This work is very important as it clearly shows some organic groups, that can be prepared using Schiff‐base chemistry, exhibit redox activity at low potentials and therefore Schiff‐base materials can be used as anodes. In their follow‐up work, the same group prepared polymeric Schiff‐bases by mixing terephthalaldehyde, *p*‐phenylenediamine, and polyether amine blocks resulting in polymers with good adhesive properties, allowing them to serve as redox‐active binders for sodium‐ion anodes.^[^ ^64^ ^]^

A series of oligomeric Schiff‐bases‐containing carboxylic end groups was investigated in a subsequent study.^[^ ^62^ ^]^ Upon treatment of these oligomers with sodium base, the sodiation of carboxylic groups occurred and sodium carboxylates formed. Although sodiated and non‐sodiated compounds behaved differently, the focus here will be on the structures and electrochemical properties of the sodiated compounds O2, O3, O4, and O5 (Figure 4B). Supported by DFT calculations, it was concluded, that NaOOC─Ar─C═N─ and ─N═C─Ar─C═N─ groups are active, while NaOOC─Ar─N═C─ and ─C═N─Ar─N═C─ groups are inactive. A linear correlation between the number of active groups and inserted Na^+^ ions were found. However, it is important to note that elimination of inactive groups and replacement with conjugated and non‐conjugated non‐aromatic linkers resulted in oligomers with worse performance. Although they do not directly contribute to the electron storage, the inactive aromatic groups are important for the overall performance of the oligomer. The authors ascribed this increase in capacity to the enhancement of the favorable π–π interactions or to reduction of N–N repulsions and the loss of planarity.

Furthermore, multiple materials based on Tp linkers were reported as anodes for sodium‐ion batteries and, although they do not contain imine bonds, they will be reviewed here as they are a direct result of Schiff‐base reaction. Lu et al. reported an anthraquinone‐containing TpCOF as sodium anode able to store up to 182 mAh g^–1^ at 50 mA g^–1^.^[^ ^65^ ^]^ Intriguingly, the redox behavior at low voltages was present although TpCOFs do not contain imine bonds that were previously shown to be crucial for low‐voltage energy storage. Based on the combination of spectroscopical investigations with DFT calculations, the mechanism of sodium storage was resolved. Firstly, at voltages around 1.5 V the anthraquinone units get reduced in a two‐step process, forming a radical intermediate upon the transfer of the first electron. At lower voltages around 0.5 V, the keto‐form of the Tp‐linker gets reduced, also following a multi‐step process involving radical intermediates. The mechanism proves TpCOFs do not store energy utilizing the same mechanism as previously described for polymers containing imine bonds. Furthermore, significant enhancement of the performance of the TpCOFs was accomplished by exfoliation, improving the capacity to more than 500 mAh g^–1^ at 50 mA g^–1^. The authors ascribed this improvement to increased radical stability in TpCOF as a consequence of reduced stacking thickness. Vaidhyanathan et al. reported a bipyridine‐tetrazine mesoporous TpCOF which exhibited very good performance with reversible capacity over 400 mAh g^–1^ at 50 mA g^–1^.^[^ ^63^ ^]^ The high capacity was ascribed to a very low unoccupied molecular orbital level—a consequence of a combination of electron‐rich and ‐deficient centers, that drive the moderate sodium kinetics. Furthermore, redox contribution of bipyridine, tetrazine, and carbonyl atoms increase the capacity (Figure 4C).

### 3.2 Lithium storage

While most of the novel materials for sodium‐ion batteries were adapted from previous work on lithium‐ion batteries,^[^ ^68^, ^69^ ^]^ this was not the case for Schiff‐base anodes at they were first employed for sodium‐ion systems.^[^ ^61^ ^]^ Nevertheless, Schiff‐base materials also perform well as anodes for lithium‐ion batteries. COFs proved as very promising materials again, and Zhao et al. synthesized an ImCOF by condensation of triformylbenzene (Tb) and 4,4′‐diamino‐biphenyl.^[^ ^70^ ^]^ The ordered material showed a capacity of over 700 mAh g^–1^ at 1 A g^–1^, along with a capacity retention of over 80% upon 500 cycles. Similarly, Zhang et al. reported another ImCOF that showed comparable performance as an anode material for lithium‐ion batteries prepared from 2,4,6‐triaminopyrimidine and terephthalaldehyde.^[^ ^71^ ^]^ Intriguingly, they utilized a simple synthetic method: grinding of the reactants and a *p*‐toluene sulfonic acid catalyst was performed with mortar and pestle and the reaction was accelerated using only a small amount of water. The mixture was then heated to 120 °C to yield the final product upon washing. This account confirms that complicated procedures utilizing variety of organic solvents are often not needed to synthesize Schiff‐base materials with good electrochemical performance. Instead, these materials can be synthesized with simple sustainable procedures.

**FIGURE 5:**
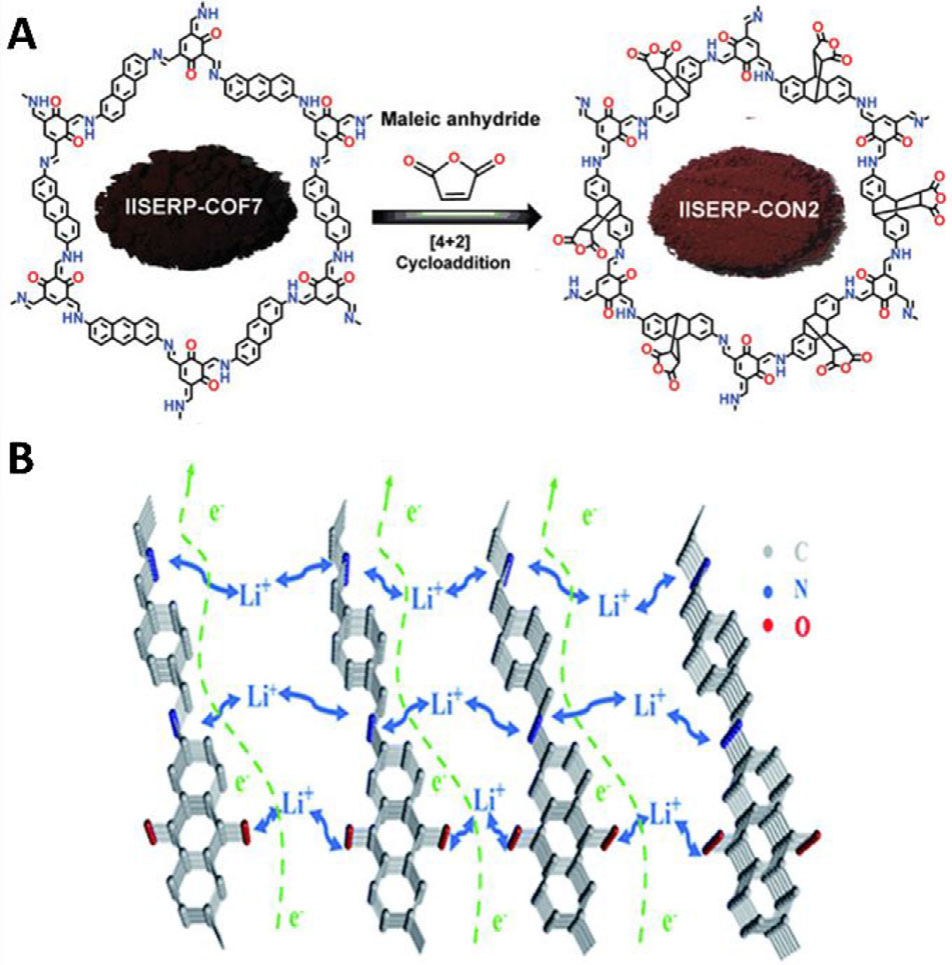
(A) Chemical exfoliation of a mixed Im/TpCOF utilizing Diels‐Alder addition of maleic anhydride. Reproduced with permission.^[^ ^66^ ^]^ Copyright 2021, Wiley‐VCH. (B) A Schiff‐base polymer containing anthraquinone units capable of storing a great amount of charge due to the variety of electroactive units. Reproduced with permission.^[^ ^67^ ^]^ Copyright 2019, The Royal Society of Chemistry

Exfoliation of COFs was able to significantly increase their performance as anodes for lithium‐ion batteries. Du et al. chemically exfoliated an ImCOF, giving a good model to quantify the performance enhancement upon exfoliation.^[^ ^72^ ^]^ The mesoporous ImCOF was exfoliated using a solution of a strong acid and a strong oxidant, namely HClO_4_ and KMnO_4_ and the restacking was prevented by the in situ formation of manganese oxide nanoparticles. The exfoliated material exhibited a capacity about 8 times greater than pristine ImCOF. The improvement was ascribed to the exfoliated structure that provided more exposed active sites and improved the lithium‐ion kinetics, while the macroporous structure provided an effective buffer to account for the volume changes during charging and discharging. A similar improvement was obtained for exfoliation of mixed Im/TpCOFs containing an anthracene unit, as reported by Vaidhyanathan et al.^[^ ^66^ ^]^ The Im/TpCOFs were exfoliated by Diels‐Alder ([4+2] cycloaddition) addition of maleic anhydride on an anthracene group (Figure 5A) that caused exfoliation and prevented restacking. However, all of these reports use rather harsh conditions for chemical exfoliation of COFs. This problem was solved by Vaidhyanathan et al. as they reported self‐exfoliating TpCOFs containing triazole moieties that form covalent‐organic nanosheets which exhibited a capacity of over 500 mAh g^–1^ at 1 A g^–1^.^[^ ^73^ ^]^

Furthermore, Zhu et al. synthesized linear Schiff polymers by simply heating a solvent‐free mixture of an aromatic diamine containing quinone groups and terephthalaldehyde at 180 °C under argon atmosphere, highlighting the possibility of sustainable synthesis.^[^ ^74^ ^]^ The resulting material exhibited a stable discharge capacity of more than 150 mAh g^–1^ at a current density of 10 mA g^–1^. Although the contribution to overall capacity was small, a reduction peak at 2.3 V and a corresponding oxidation peak at 3.2 V were observed and ascribed to quinone functionalities. Additionally, it is worth mentioning the work of Wang et al., reporting another Schiff‐base polymer anode also containing redox‐active groups, namely anthraquinone.^[^ ^67^ ^]^ Utilizing different mechanisms, every moiety was able to store up to 16 electrons (Figure 5B), thus resulting in a capacity of over 480 mAh g^–1^ at 1 A g^–1^ after 1000 cycles.

Although to our knowledge, there are no reports which explore the possibility to change the potential of the Schiff‐base redox reaction, it should be achievable via introduction of substituents. The substituents in the proximity of redox‐active groups, connected with them through conjugation, can greatly change the electronic properties of the material and subsequently change the redox potential.^[^ ^75^, ^76^, ^77^, ^78^ ^]^ Therefore, it should be possible to further tune the redox activity of Schiff‐base materials.

## 4 SCHIFF‐BASE MATERIALS WITH ADDED REDOX FUNCTIONALITIES

In the previous chapter, it has been shown that Schiff‐base materials are an intriguing option for the development of sodium‐ and lithium‐ion anodes due to the low‐voltage redox reaction of the ─N═CH─Ar─CH═N─ unit. However, many of these examples introduced additional quinone units that exhibited redox reactions at higher redox potentials. Although the Schiff‐base groups were not directly participating in this electrochemical reaction, they provided a backbone for materials containing these new redox‐active groups by incorporating them into polymeric networks and frameworks.^[^ ^63^, ^65^, ^67^, ^74^ ^]^ The possibility to introduce other redox functionalities in Schiff‐base materials significantly expands their applicability in the energy storage field, as not only a low‐potential reaction, suitable for anodes can be utilized, but also high‐potential redox reactions, suitable for cathodes. The following chapter is divided into two subchapters. The first one will review strategies to incorporate redox functionalities in the backbone of Schiff‐base materials while the second section will give an overview of the use of SNWs and frameworks as hosts for redox‐active materials.

### 4.1 Schiff‐bases as linkers for redox‐active centers

#### 4.1.1 Redox activity in aqueous electrolytes

**FIGURE 6:**
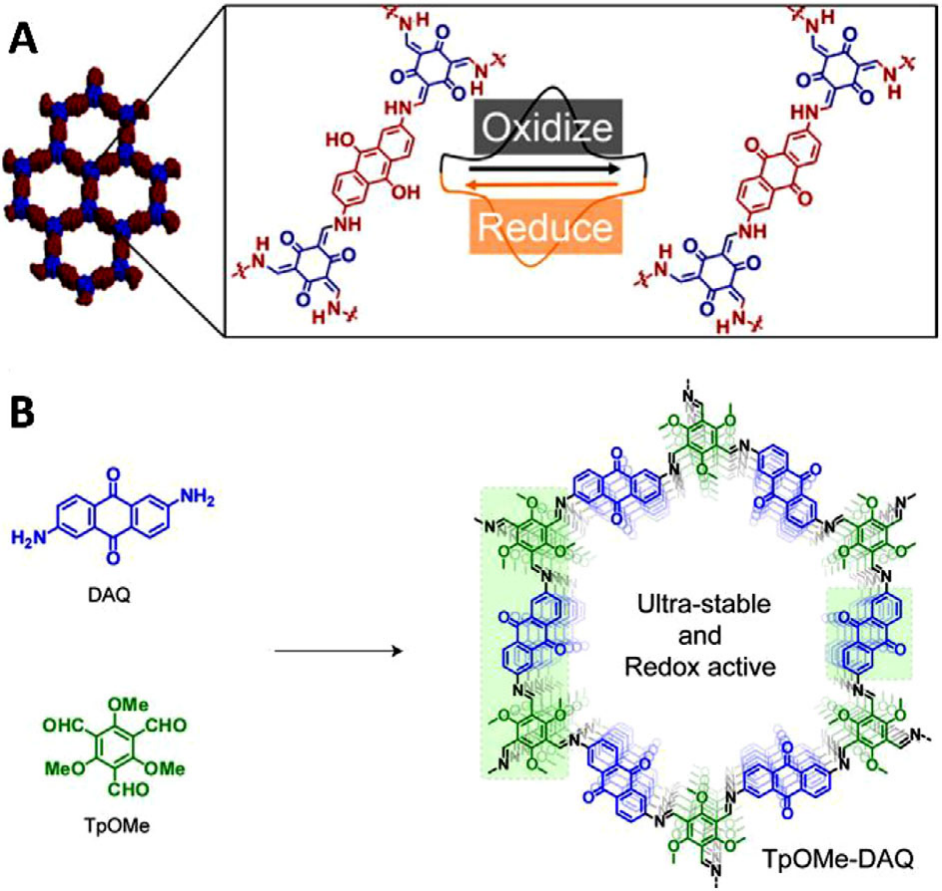
(A) Redox reactivity of an anthraquinone‐TpCOF. Reproduced with permission.^[^ ^79^ ^]^ Copyright 2013, American Chemical Society. (B) Anthraquinone‐containing ImCOF, synthesized using a methoxylated Tp linker. Adapted with permission.^[^ ^80^ ^]^ Copyright 2018, American Chemical Society

As TpCOFs, unlike Schiff‐base‐containing materials, are stable in aqueous solutions, TpCOFs decorated with redox functionalities were used for energy storage in aqueous solutions, primarily in 1 m H_2_SO_4_. A TpCOF with anthraquinone functionality, reported by Dichtel et al., was able to store around 40 F g^–1^ (Figure 6A) while a model reference, redox‐inactive TpCOF, stored only 15 F g^–1^ at a current density of 0.1 A g^–1^.^[^ ^79^ ^]^ More than 80% capacitance retention was achieved after 5000 cycles at 0.1 A g^–1^. The performance of TpCOF was further enhanced by the formation of hybrid materials with conductive additives,^[^ ^81^ ^]^ allowing for a higher amount of redox‐active centers to be reached by electrons. The hybrid materials were formed by the growth of poly(3,4‐ethylenedioxythiophene) (PEDOT), a conductive polymer in the pores of the COF,^[^ ^82^ ^]^ or by the growth of the COF on the surface of conductive carbon materials such as nanotubes,^[^ ^83^ ^]^ or graphene aerogels.^[^ ^84^ ^]^ A TpCOF containing smaller quinone functionalities, reported by Banerjee et al., which benefited from stabilization via intraCOF hydrogen bonding, showed increased capacity with 416 F g^–1^ at 0.5 A g^–1^_._^[^ ^85^ ^]^ Moreover, it showed good performance as a cathode material in aqueous zinc‐ion batteries with a performance of up to 276 mAh g^–1^ at 125 mA g^–1^.^[^ ^86^ ^]^

Dichtel et al. reported anthraquinone TpCOF thin films with a thickness up to 200 nm which showed promising performance even without conductive additives.^[^ ^87^ ^]^ The same group later reported even thicker films, which showed comparable performance upon increasing their conductivity by the formation of PEDOT in the pores of the COF.^[^ ^88^ ^]^ Similarly, Banerjee et al. reported thicker films by growing the COF on the top of carbon nanofibers.^[^ ^89^ ^]^ The same group later reported an alternative strategy to prepare thicker electroactive films, up to 200 μm, by varying the Tp linker for its methoxylated counterpart (Figure 6B).^[^ ^80^ ^]^ The possibility to increase the thickness of the films was ascribed to increased conductivity, due to the conjugation of imine groups, but the stability was not compromised due to a stabilizing effect of intraCOF hydrogen bonds. The thickness of such films is a crucial parameter for energy storage applications as the amount of electrons stored per electrode area is often relevant for applied systems.

TpCOFs are not the only type of stabilized Schiff materials that were described as electroactive materials in aqueous solutions. Liedel et al. utilized Schiff‐base chemistry to attach vanillin, a naturally occurring guaiacyl‐containing aldehyde, on chitosan, a naturally occurring polysaccharide containing amine groups.^[^ ^90^ ^]^ The formed material showed no redox activity in 1 m HClO_4_, probably due to the fast hydrolysis of the imine groups. However, reduction of the imines using sodium cyanoborohydride, a selective reduction agent for imines, yielded a redox‐active material with good stability in the aqueous electrolyte that exhibited a capacity of around 90 mAh g^–1^ at 0.1 A g^–1^.

It is worth mentioning that while many redox‐active Schiff materials are often called supercapacitors in the literature, this is not necessarily a correct classification. Supercapacitors store electrons either through an electric double layer or through pseudocapacitance, a surface redox phenomenon characteristic for metal oxides often mistakenly ascribed to redox‐active organic polymers.^[^ ^2^, ^91^ ^]^

#### 4.1.2 Redox activity for alkali‐ion batteries

**FIGURE 7:**
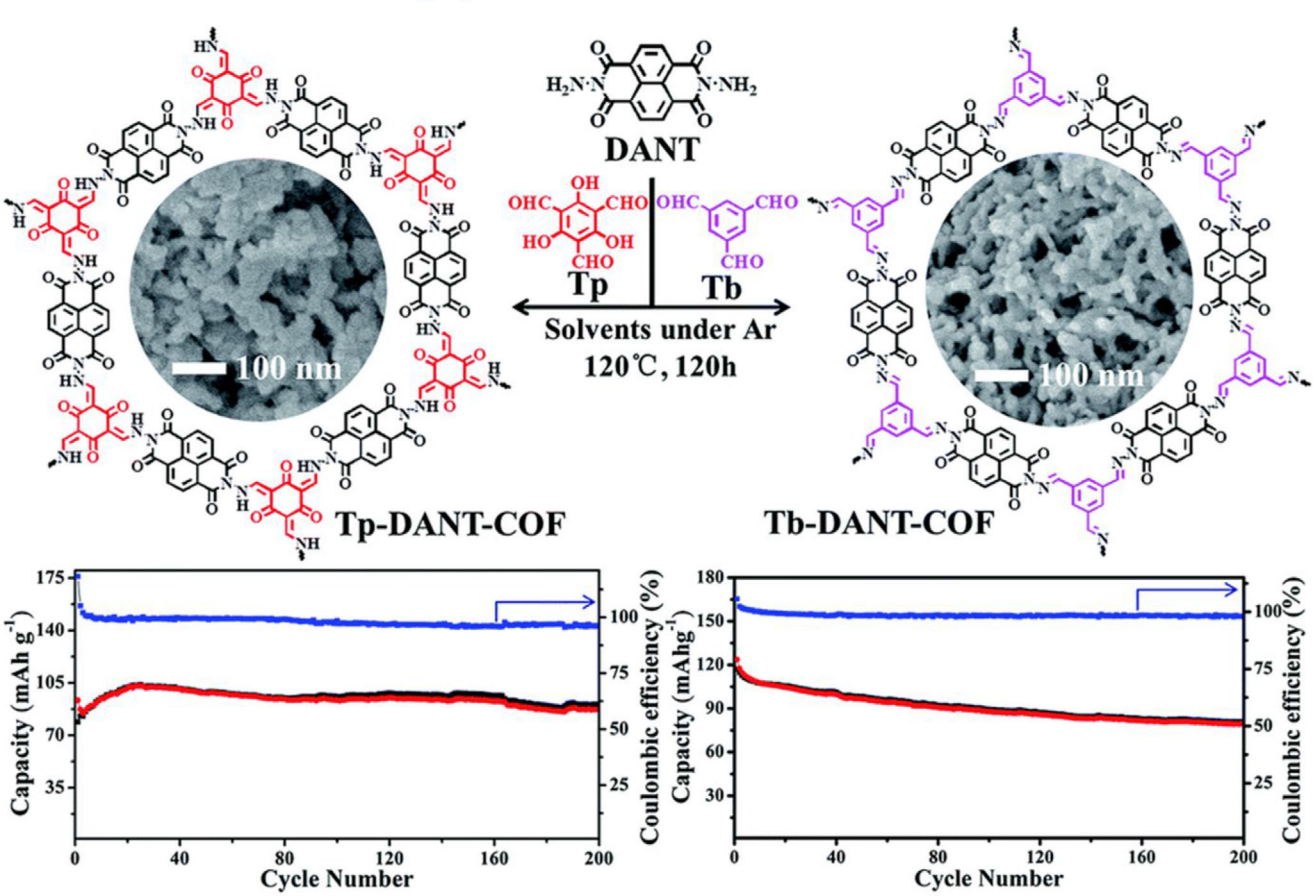
Two COFs prepared with the same redox‐active diamine linker but different trialdehyde nodes resulting in a TpCOF and a ImCOF and their electrochemical performance at around 0.2 A g^–1^ (red—discharge, black—charge). Reproduced with permission.^[^ ^92^ ^]^ Copyright 2016, The Royal Society of Chemistry

Bu et al. reported the first imine‐based COFs as cathode material for lithium‐ion batteries.^[^ ^92^ ^]^ The COFs were based on a naphthalimide, redox‐active moiety, containing diamine and a trialdehyde linker, either Tp or Tb, yielding TpCOF and ImCOF. Intriguingly, the ImCOF showed better performance than the TpCOF. The difference was ascribed to the conjugated structure that eased the electron transport in the materials (Figure 7). This result is very important as it shows that the stability of TpCOFs does not play a crucial role in aqueous‐free systems and therefore confirms that Schiff‐base materials can be used in (nonaqueous) alkali‐ion batteries. Furthermore, usage of TpCOFs, as opposed to ImCOFs, can negatively impact the performance of the formed materials, while severely limiting the library of aldehydes usable in these materials.

As seen previously for anodes, the performance of COF‐based cathodes for lithium‐ion batteries can also be increased by exfoliation. Wang et al. exfoliated an anthraquinone‐containing TpCOF in a ball mill. Although the performance of the exfoliated COF was similar to the performance of the pristine COF at low current densities (50 mA g^–1^), it differed greatly at higher current densities.^[^ ^93^ ^]^ For example, it stored three times as much lithium at 3 A g^–1^. This difference has been ascribed to eased diffusion pathways. Furthermore, the exfoliated COF exhibited great capacity retention, exhibiting a capacity of over 100 mAh g^–1^ even after 1800 cycles at 0.5 A g^–1^. Additionally, similar results were obtained for other TpCOFs and ImCOFs. Moreover, the formation of a hybrid material with conductive additives proved to be an effective way to increase the performance of ImCOFs. For example, growth of conductive polymer in their pores^[^ ^94^ ^]^ or synthesis of the TpCOF on the surface of conductive carbon nanotubes (CNTs)^[^ ^95^ ^]^ have been reported as promising strategies for that purpose.

Liedel et al. attached aldehydes bearing different redox‐active and inactive groups to polyallylamine, a commercial polymer rich in primary amine groups, using Schiff‐base chemistry in ethanol at room temperature.^[^ ^96^ ^]^ The formed Schiff‐base polymers were used as model compounds to study the electrochemical activity of catechol groups in lithium‐ion batteries and compared the performance to redox inactive isomeric resorcinolic groups. Finally, the catecholic baring polymer exhibited a capacity of over 100 mAh g^–1^ at 0.05 A g^–1^, approximately 20% of which was ascribed to the redox‐reaction of catecholic groups. The authors showed that this strategy can be applied to other aldehydes, providing an interesting platform for the development of new materials containing different redox active groups.

### 4.2 Schiff‐bases frameworks and networks as hosts for redox‐active materials

**FIGURE 8:**
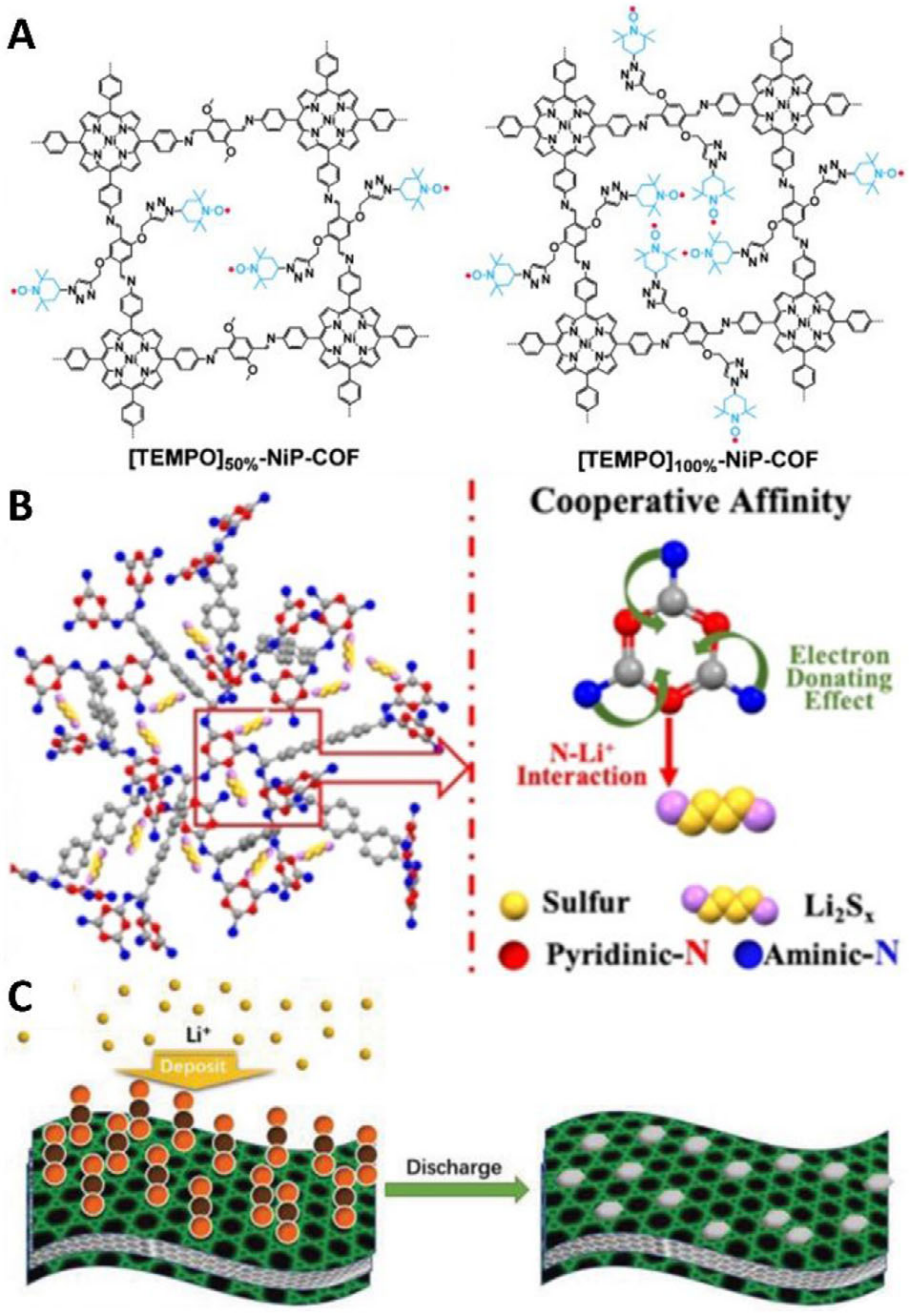
(A) Functionalization of ImCOFs with 2,2,6,6‐tetramethyl‐1‐piperidinyloxy functionalities. Reproduced with permission.^[^ ^97^ ^]^ Copyright 2015, Wiley‐VCH. (B) Schiff‐base networks as hosts for sulfur in lithium‐sulfur batteries. Reproduced with permission.^[^ ^98^ ^]^ Copyright 2021, IOP Publishing, Ltd. (C) Discharging of a Li‐CO_2_ battery with a CO_2_ enriched COF due to nanoconfinement. During discharge, lithium carbonate is formed that fills the pores of the COF. Reproduced with permission.^[^ ^99^ ^]^ Copyright 2019, Wiley‐VCH

Redox‐inactive Schiff materials allow for introduction of redox‐active functionalities by post‐synthetic modification. For example, Jiang et al. reported ethynyl group‐containing ImCOFs which were post‐synthetically modified via click‐reaction introducing 2,2,6,6‐tetramethyl‐1‐piperidinyloxyl (TEMPO) functionalities (Figure 8A), able to store one electron.^[^ ^97^ ^]^ For every terephthalaldehyde linker one ([TEMPO]_50%_‐NiP‐COF) or two ([TEMPO]_100%_‐NiP‐COF) TEMPO units were added. While both materials exhibited redox activity in an organic electrolyte, [TEMPO]_100%_ showed better performance due to the higher density of TEMPO groups. However, such a high density of functional groups hinders the transportation of ions inside the pores and therefore [TEMPO]_50%_ exhibited better diffusion properties and in consequence better rate stability. While [TEMPO]_100%_ performed significantly better than [TEMPO]_50%_ at low current densities (167 F g^–1^ as compared to 124 F g^–1^ at 0.1 A g^–1^), the difference was less significant at high current densities (113 F g^–1^ as compared to 101 F g^–1^ at 2 A g^–1^).

Sulfur is one of the most commonly used materials to introduce redox activity in Schiff‐base materials. Sulfur is an inexpensive byproduct of the chemical industry. It is redox‐active at relatively high potentials with a theoretical capacity of 1672 mAh g^–1^, which makes it an attractive choice for commercial lithium‐ion batteries. However, there are many challenges for the commercialization of lithium‐sulfur batteries. Significant volume changes during cycling can pulverize the electrode. Loss of active material due to the dissolution of polysulfide intermediates causes abrupt decay in the capacity. Shuttling of the dissolved species can significantly reduce capacity retention as shuttling species recombine on the anode.^[^ ^100^ ^]^ However, embedding the sulfur in nitrogen‐rich networks can reduce these effects, making polymeric Schiff‐bases an exciting solution for lithium‐sulfur battery cathodes.

Zhang et al. reported a mesoporous TpCOF containing azo‐groups which was impregnated with molten sulfur and investigated as a cathode material for lithium‐sulfur batteries.^[^ ^101^ ^]^ The impregnation of sulfur in the mesopores was testified by the lack of sulfur particles as observed by SEM imaging, lack of sulfur reflections in XRD due to nanoconfinement, and improved thermal stability of the sulfur within the COF composite. Even without the use of further electrolyte additives as commonly used in lithium‐sulfur batteries, this cathode material delivered a high initial discharge capacity of 1536 mA g^–1^ and capacity retention of 741 mAh g^–1^ after 100 cycles at 0.1 C (where C is the theoretical capacity of sulfur). The material showed excellent rate capability, ascribed to the mesoporous structure of the composite. Later, many other COFs were reported for lithium‐sulfur batteries containing different moieties such as ImCOFs containing porphyrin^[^ ^102^ ^]^ and pyrene moieties.^[^ ^103^ ^]^ A lot of interesting strategies were exploited to increase interaction between the sulfur and the COF. A vinyl‐containing COF was synthesized by Chen et al. and the sulfur was attached to it by inverse vulcanization reaction on the vinyl bonds.^[^ ^104^ ^]^ Additionally, Zhang et al. reported a fluorine‐rich COF which was used to facilitate nucleophilic substitution reaction that resulted in sulfur being anchored to the framework.^[^ ^105^ ^]^ Mak et al. reported another approach that utilized cationic COFs formed from ethidium bromide. Upon introduction of polysulfide anions by anionic exchange, the sulfur easily deposited on the surface of the cationic COF.^[^ ^106^ ^]^ Additionally, COF‐functionalized membranes were successfully used to prevent the shuttling polysulfides from crossing from the cationic to the anionic part of the battery, as reported by Cai et al.^[^ ^107^ ^]^ These accounts testify the importance of the design of the bond between the sulfur and the sulfur host material.

Not only COFs but also porous Schiff‐based networks synthesized from melamine and dialdehyde provided a good platform as hosts for sulfur in lithium‐sulfur batteries, as reported by Li et al.^[^ ^98^ ^]^ (Figure 8B). Networks were synthesized using a simple microwave‐assisted catalyst‐free approach in DMSO and subsequently loaded with sulfur. The network synthesized from 4,4′‐biphenyldicarboxaldehyde exhibited the best performance retaining a capacity of 620 mAh g^–1^ at 0.5 C after 500 cycles. Nevertheless, terephthalaldehyde‐based SNW retained 310 mAh g^–1^ at 0.5 C after 500 cycles. In view of the low price of the precursors, this can be seen as a remarkable performance.

COFs were used not only for Li‐sulfur batteries but also for Li‐CO_2_ batteries where carbon dioxide works as a cathode material and gets reduced during discharge forming Li_2_CO_3_ and carbon. Meng et al. reported a COF host with a dual purpose: firstly it increased the concentration of CO_2_ on the cathodes of Li‐CO_2_ batteries which, as predicted by the Nernst equation, increased the discharge voltage of the battery.^[^ ^99^ ^]^ Secondly, the COF prevents the formation of large crystallites of Li_2_CO_3_ and therefore eases the charging process (Figure 8C). Similarly, Loh et al. reported an ImCOF hybridized to ruthenium nanoparticle‐coated CNTs which proved to be a highly efficient cathode for the Li‐CO_2_ battery.^[^ ^108^ ^]^ To our knowledge, there is a lack of research on sustainable SNWs for Li‐CO_2_ batteries but, analogous to Li‐sulfur batteries, they hold a great promise.

## 5 MICROPOROUS SCHIFF‐BASE MATERIALS FOR SUPERCAPACITORS

COFs and SNWs are porous materials with high surface areas making them attractive options for supercapacitor applications, but they suffer from low conductivity. However, the conductivity problem can be minimized by growing the ImCOF on top of for instance amine‐functionalized graphene forming a hybrid material with enhanced conductivity. The graphene serves as an integrated current collector, allowing electrons to pass small distances through the low‐conductivity COFs to high‐conductivity graphene sheets, thus addressing the challenge of limited conductivity. Intriguingly, the formed hybrid material showed good performance as a supercapacitor in 1 m sodium sulfate aqueous solution exhibiting capacitance of 533 F g^–1^ at 0.2 A g^–1^, as reported by Sun et al., even though the imine bonds were present in the hybrid material.^[^ ^111^ ^]^ The surprising resistance of the imine bonds to hydrolysis could be ascribed to the pH neutral nature of the electrolyte, thus hindering degradation. Moreover, a stabilizing effect of graphene sheets, possibly through π‐π interactions, was proposed. However, low stability compared to other supercapacitor materials, retaining only 79% of the capacitance upon 1000 cycles, can be ascribed to hydrolysis of the imine bonds.

**FIGURE 9:**
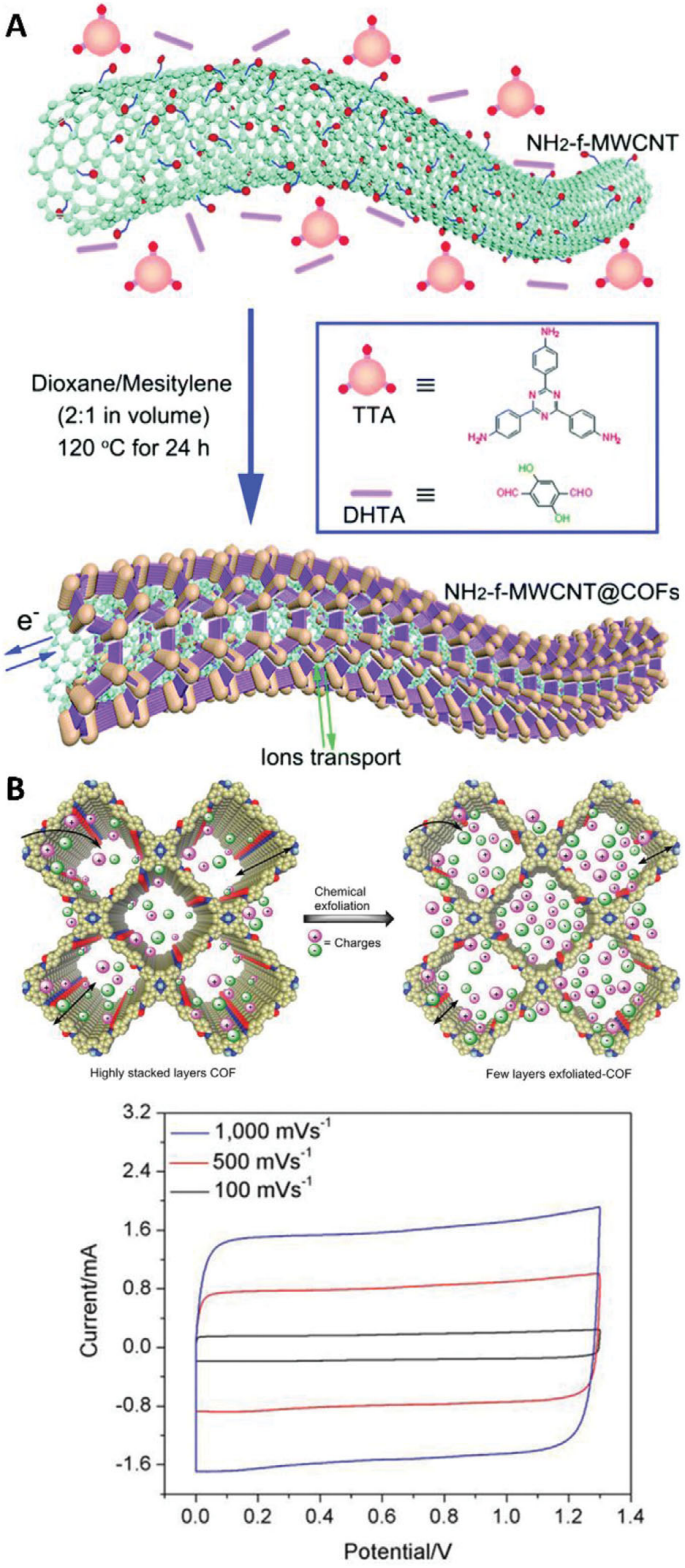
COFs for supercapacitors. (A) ImCOF grown on the surface of multiwalled carbon nanotubes. Reproduced with permission.^[^ ^109^ ^]^ Copyright 2017, The Royal Society of Chemistry. (B) Exfoliated ImCOF with corresponding cyclic voltammetry at different scan rates. Reproduced with permission.^[^ ^110^ ^]^ Copyright 2021, Wiley‐VCH

Stabilization of COFs can improve capacity retention. Wang et al. created a COF by a reaction of a triamine with 2,5‐dihydroxyterephthalaldehyde (Figure 9A) forming a framework stabilized by, as described previously,^[^ ^112^ ^]^ intramolecular hydrogen bonding.^[^ ^109^ ^]^ The COF was grown on the surface of amine‐functionalized multiwalled CNTs (MWCNTs), forming a highly conductive hybrid material that retained 96% of the capacitance after 1000 cycles in the same electrolyte as described before, probably due to the higher stability of the formed COF. Additionally, the importance of the amine functionalization of MWCNTs was discussed as the hybrid material formed with non‐functionalized MWCNTs performed much worse than the one with functionalized ones. This work points to the importance of the design of the carbon additive – Schiff base material interface. Bhaumik et al. reported a COF with a Tp‐like linker based on diaminonaphthalene which stored 348 F g^–1^ at 0.5 A g^–1^ in diluted sulfuric acid even without the formation of a hybrid material with a more conductive carbon.^[^ ^113^ ^]^ Similar to previous reports, exfoliation of COFs proved to be beneficial for the formation of supercapacitors and an exfoliated mesoporous COF showed truly rectangular cyclic voltammograms, characteristic for supercapacitors (Figure 9B).^[^ ^110^ ^]^

## 6 SCHIFF‐BASE POLYMERS AS PRECURSORS FOR CARBON MATERIALS

**FIGURE 10:**
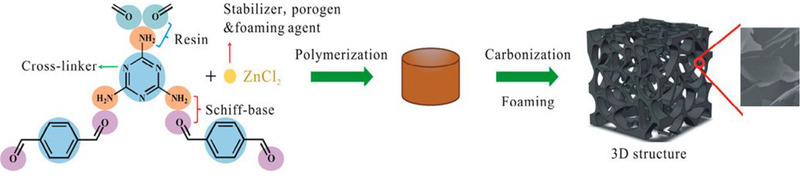
Synthetic process to obtain Schiff‐base derived carbons. It is possible to vary the chemical nature of the cross‐linker (blue), the geometry of amine/aldehyde groups (orange/violet), the carbonization temperature, as well as the use/absence of different porogens (yellow). Reproduced with permission.^[^ ^114^ ^]^ Copyright 2018, American Chemical Society

The ease of Schiff‐base chemistry renders the great variety of possible products as versatile and suitable precursors for the synthesis of porous carbon materials (Figure 10). Since ideal precursors for carbonization processes necessarily should not evaporate or sublime at elevated temperatures, larger molar masses of the precursor molecules are generally beneficial and in turn, lead to increased carbonization yields in most cases.^[^ ^33^, ^115^ ^]^ Here, Schiff‐base polymers come into play as they have relatively large molar masses. Moreover, carbons prepared from Schiff‐base materials are inherently nitrogen‐doped, making them suitable for many applications.^[^ ^38^ ^]^ The variety of precursors and a defined carbonization mechanism offer a great scientific playground. Schiff‐base polymers allow for a precisely controllable carbon‐precursor synthesis and thus, enable the synthesis of carbons with tuneable properties. In recent years, a rich variety of such carbonaceous materials was synthesized. It is possible to distinguish or classify these materials in different ways—here we will emphasize the chosen synthetic route. Moreover, we will distinguish between reports using the pristine carbon and approaches where electroactive elements/molecules or substrates have been added to enable or foster the desired application.

Most electrochemical energy storage applications require a high surface area carbonaceous material to either increase the accessible surface area for electrosorption processes (namely supercapacitors)^[^ ^116^ ^]^ or to increase the density and/or accessibility of redox‐active sites (namely batteries).^[^ ^117^ ^]^ Taking these considerations into account, the vast majority of synthetic protocols makes recourse to well‐established porogens, such as ZnCl_2_, KOH, or K_2_CO_3_, which support the development of pronounced porosity in the resulting Schiff‐base‐derived carbons during the ongoing carbonization process.^[^ ^115^ ^]^ Approaches without porogen addition resulted in materials with modest porosity.^[^ ^118^ ^]^

Probably one of the most straightforward and facile approaches to synthesize carbon materials from Schiff‐base precursors has been published by Carriazo et al. By conducting a one‐pot synthesis, highly activated carbons from melamine and terephthalaldehyde could be synthesized.^[^ ^119^ ^]^ The polymerization and subsequent carbonization of melamine and terephthalaldehyde in the presence of an eutectic mixture of NaOH/KOH allowed for the synthesis of high specific surface area carbons (up to 3300 m^2^ g^–1^) that could be applied as electrodes for supercapacitors. These materials showed good performance using 6 m KOH as electrolyte. The authors report on specific capacitance values of more than 250 F g^−1^ that could be achieved even at a high current density of 10 A g^−1^.

A sustainable protocol using biomass waste as a precursor to generate an imine‐bonded carbonization precursor was introduced by Borchardt et al.^[^ ^120^ ^]^ Using mechanochemistry under solvent‐free conditions, lignin, urea, and potassium carbonate as porogen were used to synthesize polymers with imine‐connected domains. An in‐depth optimization study to find the best precursor for the final carbonization led to materials displaying very high specific surface areas up to 3000 m^2^ g^–1^ as well as a high total pore volume of 2 cm^3^ g^–1^. Owing to these very high surface areas, these materials proved to be well suited for electrochemical energy storage as supercapacitor electrodes. In their contribution, the resulting N‐doped carbons were tested in aqueous (1 m Li_2_SO_4_ electrolyte: 177 F g^−1^), organic (1 m tetraethylammonium tetrafluoroborate in MeCN: 147 F g^−1^) as well as ionic liquid electrolytes (1‐ethyl‐3‐methylimidazolium tetrafluoroborate: 192 F g^−1^), and displayed high specific capacitances.

Comparable approaches using ZnCl_2_, serving as solvent and porogen, but with different monomers were published by Cai et al. (conjugated polyquinoneimine derived network),^[^ ^121^ ^]^ Gan et al. (melamine, terephthalaldehyde, formaldehyde),^[^ ^114^ ^]^ and Xiong et al. (melamine, terephthalaldehyde, pyrrolidine).^[^ ^122^ ^]^ All reports aimed for the incorporation of a high amount of nitrogen as well as oxygen in the resulting carbon materials. To this end, carbonaceous polymers were synthesized at relatively low pyrolysis temperatures ranging between 500 and 750 °C. In consequence, very high oxygen contents of more than 20 wt% (Cai et al.), 10 wt% (Gan et al.), and 7 at% (Xiong et al.) were achieved. Owing to low carbonization temperatures, the resulting materials were predominantly microporous, at least at temperatures up to 700 °C. However, Xiong et al. received micro/mesoporous Schiff‐base derived carbons. In turn, the impact of the pore size as well as of the heteroatom amount could be investigated with respect to the applied pyrolysis temperature. While Cai et al. could achieve capacitances as high as 479 F g^−1^ at 0.1 A g^−1^ and 125 F g^−1^ at 10 A g^−1^ in 1 m H_2_SO_4_, Gan et al. used an alkaline electrolyte to achieve 268 F g^−1^ at 2.0 A g^−1^ in 6 m KOH. The materials of Xiong et al. performed well in both acidic and alkaline electrolytes, displaying 377 F g^−1^ at 0.2 A g^−1^ in 1 m H_2_SO_4_ and 300 F g^−1^ at 0.1 A g^−1^ in 6 m KOH.

**FIGURE 11:**
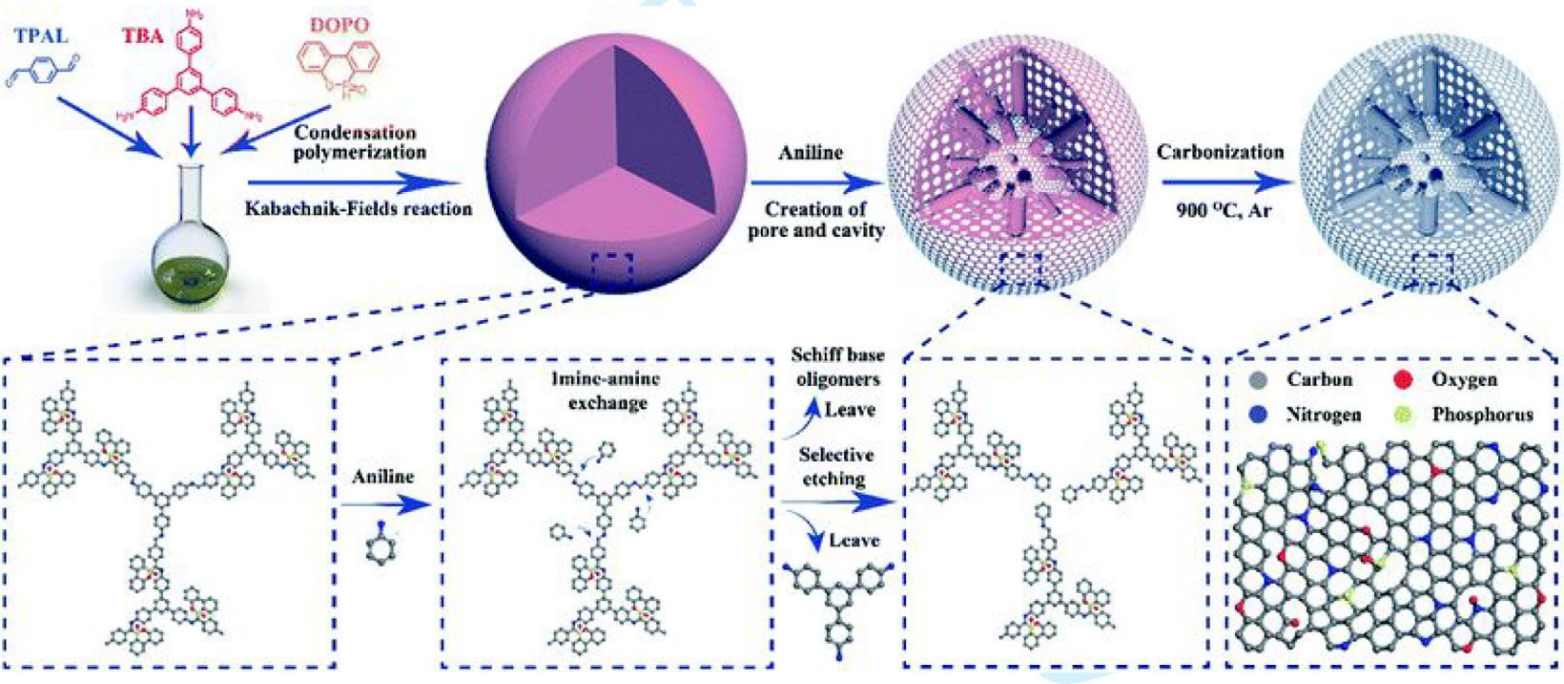
Formation of hierarchical multi‐heteroatom‐doped (N, O, P) carbon nanospheres using an etching approach. Reproduced with permission.^[^ ^124^ ^]^ Copyright 2020, The Royal Society of Chemistry

Spherical Schiff‐base precursor‐derived carbon materials for application as supercapacitor electrodes were investigated in the works of Chao et al. and Dai et al.^[^ ^123^, ^124^ ^] ^The methodology reported by Chao et al. entails the polymerization of *p*‐terephthalaldehyde and 2,6‐diaminopyridine and subsequent KOH activation to yield N‐doped carbon nanospheres. Evidently, an optimized porosity of the materials (809 m^2^ g^–1^) led to the highest specific capacitances (732 F g^−1^ at 8 A g^−1^) in 6 m KOH electrolyte. Without any porogen, Dai et al. succeeded in synthesizing multi‐heteroatom‐doped (N, O, P) carbon nanospheres (Figure 11). Thus, this group tried to tackle one aspect of structural optimization for the intended application—namely the chemical nature of the pore (surface polarity) by heteroatom doping. In parallel, the materials received also show micro/mesoporosity. A reasonable argumentation for true hierarchy is stated by thermal etching/cleavage of oligomeric/low mass amine intermediates as a result of imine reorganization upon the addition of aniline. Carbonization without the need for a porogen yielded hierarchical porous carbon nanospheres. These materials performed well in acidic conditions using 1 m H_2_SO_4_ as electrolyte (359 F g^−1^ at 0.5 A g^−1^). Interestingly, semi‐solid‐state supercapacitors could be assembled using the carbon spheres and H_2_SO_4_/polyvinyl alcohol as a gel electrolyte.

A very unconventional approach towards the synthesis of high surface area materials for supercapacitors that also circumvents the need for porogens was published by Liao et al.^[^ ^125^ ^]^ A sol‐gel approach was applied to synthesize a Schiff‐base porous organic polymer aerogel followed by subsequent pyrolysis. The resulting materials display comparably high surface areas of up to 2350 m^2^ g^–1^ and a low bulk density of 5 mg cm^–3^ as a result of the underlying gelation process. The so‐obtained materials display high specific capacitance, 300 F g^−1^ at 0.5 A g^−1^, and a fast charge rate. Charging to 221 F g^−1^ was possible within only 17 s.

All the materials presented up to now were directly used as electrode materials without adding further functionality to the materials via molecular chemical engineering. Feng et al. covalently coupled a Schiff‐base 2D porous polymer (from melamine and aromatic dialdehydes) onto graphene sheets to obtain a hybrid material that benefits from the structural features of both building blocks (N‐doping of polymer, conductivity of graphene).^[^ ^126^ ^]^ Their strategy includes coupling of melamine and aromatic dialdehydes to aminated graphene oxide layers—thus serving as structure‐dictating template. Concomitant polymerization of melamine and aldehydes led to Schiff‐base polymers coupled onto graphene oxide. Upon pyrolysis, carbonaceous hybrid materials were obtained showing exceptionally high capacitances of up to 424 F g^−1^ at 0.1 A g^−1^ in 6 m KOH, clearly excelling the same porous carbons without graphene involved.

**FIGURE 12:**
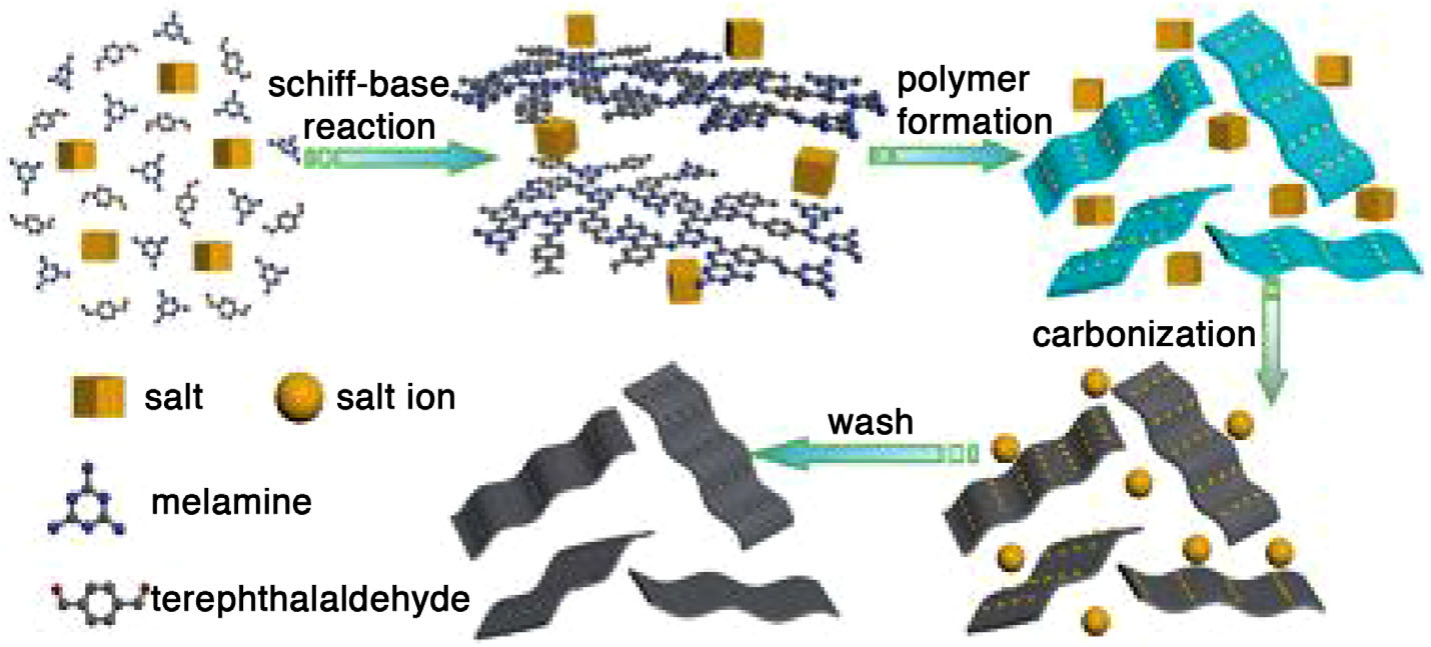
Synthesis of carbon nanosheets using eutectic salt mixtures of LiCl/KCl. Reproduced with permission.^[^ ^127^ ^]^ Copyright 2015, The Royal Society of Chemistry

All the synthetic pathways that have been illuminated yet strived for application as electrode materials in supercapacitors. In contrast, Schiff‐base derived carbon materials were also intensely studied for other applications such as different batteries (lithium‐ion,^[^ ^127^ ^]^ lithium‐sulfur,^[^ ^128^ ^]^ sodium‐ion,^[^ ^129^ ^]^ zinc‐air^[^ ^130^ ^]^) as well as capacitive deionization,^[^ ^131^ ^]^ a deionization technology that relies on comparable requirements as supercapacitors, and even electrocatalysis, such as oxygen reduction reaction.^[^ ^132^ ^]^ A salt‐templating strategy has been pursued by Lu et al.^[^ ^127^ ^]^ They synthesized a series of porous carbon nanosheet materials (Figure 12) using melamine and terephthalaldehyde as carbon precursors in molten salt medium (LiCl–KCl). Through variation of the respective amount of the individual salt fractions, it becomes possible to tune the porosity of the resulting carbon materials. The main impact of the salt medium can thereby be ascribed to the mixing/demixing behavior of the respective polymeric species in the salt mixture as a result of different solubility when varying the salt fraction. Analogous to graphite, the resulting carbon nanosheets were used as anode materials in lithium‐ion batteries (LIB). Those materials exhibit an initial coulombic efficiency of ca. 63 % (thus, being relatively high for a porous material), and a high and constant reversible capacity of 605 mAh g^−1^ at a current density of 100 mA g^−1^ even after 100 cycles. This excellent electrochemical behavior is explained with the high nitrogen content (30 wt%) and the favorable 2D structure of the material. Recently, a one pot synthesis and carbonization of partially condensed polymers synthesized from melamine and terephthalaldeheyde proved to be a facile strategy for synthesis of N‐doped hard carbons. These materials showed a temperature dependent evolution of porosity, thus enabling the synthesis of carbons containing closed pores which are hardly accessible for solvent molecules. This feature renders the materials attractive for application as anode in sodium‐ion batteries, since such closed pores are ideally suited to store quasi‐metallic sodium species.^[^ ^133^ ^]^ The best performing carbon, synthesized at 1000 °C, performed well as sodium anode, being able to store up to 180 mAh g^–1^ at (C/20) rate.

In 2018, Zhi et al. reported the use of CNT‐supported Schiff‐base‐derived carbons as cathode materials in lithium‐sulfur batteries.^[^ ^128^ ^]^ A physical mixture of melamine and terephthalaldehyde was mixed with CNTs in solution. Upon polymerization and carbonization without additional porogen, the authors proved the growth of nitrogen‐rich domains on the CNT backbone. This strategy provides conductivity and serves for the later purpose as cathode material. After melt impregnation of sulfur, the final cathode material is obtained and displays excellent cycling stability with a specific capacity of 729 mAh g^−1^ even after 500 cycles at 1 C. These results support the assumption that sulfur leaching via polysulfide shuttle mechanism, and thus capacity loss, can be effectively suppressed by trapping these species using polar nitrogen‐rich carbons. The ability to retain polysulfide species is concluded to be the main reason for the constant cycling behavior.

An interesting report was published by Lin et al. in 2021.^[^ ^130^ ^]^ The group synthesized triazine‐containing imine‐linked precursors. After infiltration with cobalt acetate, the precursor was pyrolyzed to receive Co‐doped nitrogen‐rich carbon materials. XPS analysis revealed, that the final carbon materials contain approximately 3.6 at% cobalt in various bonding motifs. Cobalt is a suitable electrocatalyst for the oxygen reduction reaction (ORR), occurring at the cathode side of Zn‐air batteries and it was assumed that cobalt, in the prepared Co@carbon material, promoted the ORR at the cathode. Another approach to synthesize efficient catalysts for the ORR was reported by Tan et al.^[^ ^132^ ^]^ They describe a strategy to prepare heteroatom (N, P) doped carbon materials by pyrolysis of imine‐linked porous organic polymers that contained high amounts of both phosphorus and nitrogen. The so‐prepared materials show high surface areas of up to 1500 m^2^ g^−1^ and pore hierarchy as well as an excellent performance in the ORR. Thus, without metal co‐doping but instead using a dual‐doping strategy (P, N), they succeeded in synthesizing high performance electrocatalysts.

## 7 OUTLOOK

Materials containing Schiff‐base groups are a promising platform for the development of organic electrode materials for batteries and supercapacitors. The conjugated nature and high molecular weight of these materials make them highly insoluble in electrolytes, resulting in good stability. The ease of their synthesis, which can be performed under solvent‐free conditions or in benign solvents, such as water, makes them ideal candidates for sustainable energy storage of the future. Furthermore, the simplicity of their synthesis can help to expand the base of researchers investigating organic electrode materials as Schiff‐base materials can be synthesized without the usage of specialized equipment for organic synthesis, making them accessible to a wide range of researchers in the energy storage community.

The application of Schiff‐base materials is limited by their instability when exposed to humidity, but this does not represent a problem for the application in alkali‐ion batteries as these batteries necessarily require water‐free conditions. Furthermore, Schiff‐base materials can also be additionally stabilized to withstand aqueous media by either reducing their content of imine groups or stabilizing them by exploiting keto‐enol tautomerism and/or intramolecular hydrogen bonding. However, these stabilization techniques often reduce the conjugation of the networks, sometimes even reducing the electrochemical energy storage properties of these materials, as described in the case of Li‐ion cathodes. In our opinion, a substantial proof that stabilized Schiff‐base materials exhibit higher stability in aqueous free systems is still not available. Therefore, it should be reconsidered if Schiff‐base materials applied in an aqueous‐free environment profit from stabilization. Stabilization not only reduces their performance, but concomitantly makes the synthesis of the materials more complicated by introducing additional steps and/or limiting the choice of precursors.

As can be seen from this short review, the field of Schiff‐base materials utilized for energy storage is dominated by crystalline and microporous COFs. These materials are usually prepared using elaborate synthetic procedures from a rather limited library of precursors involving organic solvents and extensive washing steps. While the benefit those ordered materials give to the research community to understand the structure‐property relationship is undeniable, they also hinder the commercial interest due to their often complicated synthetic procedures and costly precursors. Nevertheless, this review shows that different amorphous materials prepared from low‐cost precursors and with rather simple synthetic procedures can be utilized for energy storage applications just as effectively as many COFs. At this moment, most of the applications in the electrochemical energy storage field are covered both by crystalline COFs and amorphous Schiff‐base materials, however, there are still some applications not covered by low‐cost sustainable Schiff‐base materials and hence, there is a need for further exploration of such compounds. These low‐cost Schiff‐base materials do not only outperform their crystalline counterparts in terms of price, but also a variety of other functional materials. Other covalent‐organic frameworks, as well as metal‐organic frameworks and porous polymers, suffer from the high price of the precursors and complicated synthetic methods. Therefore, we envision low‐cost Schiff‐base materials could take the spotlight from other functional materials in the industrial context. Furthermore, we are not aware of any other low‐cost and sustainable precursors for carbonaceous materials with such a high level of nitrogen doping (even higher than 30%).

Future research efforts should be oriented towards the synthesis of new ordered materials for a better understanding of the underlying mechanisms of energy storage. In addition, expanding the library of novel materials using inexpensive precursors and procedures is of paramount interest. We want to emphasize the importance of melamine‐based materials, which is as such a rather low‐cost chemical widespread in the chemical industry. The large variety of small molecules containing multiple aldehyde groups could yield Schiff‐base materials with different organic motifs. Furthermore, the carbonization of such materials will yield nitrogen‐doped carbons, which also hold promise due to the possibility of co‐doping using the precursors which contain other heteroatoms, such as sulfur and phosphorus. Schiff‐base materials cover a wide range of properties that can be tuned by changing the precursors, we expect this to be utilized in the formation of core‐shell Schiff base materials which contain variety of different properties. Furthermore, synthetic method should be developed to combine carbonized Schiff‐bases with the pristine ones, creating materials that have maximum benefit from the addition of their individual properties.

## CONFLICT OF INTEREST

The authors declare no conflict of interest.
